# Magnetic resonance imaging and thallium-201 scintigraphy for the diagnosis of localized pigmented villonodular synovitis arising from the elbow: A case report and review of the literature

**DOI:** 10.3892/etm.2013.1022

**Published:** 2013-03-21

**Authors:** KAZUTAKA KOTO, HIROAKI MURATA, TOMOYA SAKABE, TAKAAKI MATSUI, NAOYUKI HORIE, YASUSHI SAWAI, YOSHIRO TSUJI, TOSHIKAZU KUBO

**Affiliations:** Department of Orthopedics, Graduate School of Medical Science, Kyoto Prefectural University of Medicine, Kamigyo-ku, Kyoto 602-8566, Japan

**Keywords:** magnetic resonance imaging, thallium-201 scintigraphy, pigmented villonodular synovitis, localized type, elbow joint, soft tissue tumor

## Abstract

Pigmented villonodular synovitis (PVNS) arising from the elbow joint is extremely rare; only 24 cases have been reported. It is extremely difficult to differentiate PVNS from other soft tissue tumors on the basis of imaging findings alone. Therefore, a biopsy is required for definitive diagnosis. A 20-year-old female reported a mass on her right elbow. Physical examination revealed a tumor measuring 3.0x3.0 cm. Magnetic resonance imaging (MRI) revealed that the signal intensity of the tumor was isointense to muscle on T1-weighted images; however, it was hyper- or isointense to muscle on T2-weighted images. In images obtained by gadolinium-enhanced MRI, the margin of the tumor was well-contrasted. Thallium (Tl)-201 scintigrams revealed an abnormal accumulation in the area of the mass in the early and delayed phases. On the basis of clinical findings, imaging characteristics and incision biopsy results, localized PVNS was diagnosed and marginal excision was performed. We thus identified an extremely rare case of PVNS arising from the elbow joint. When interpreting Tl-201 images for the assessment of bone and soft tissue lesions, it is important to recognize PVNS as a condition that simulates malignant tumors. Furthermore, PVNS should be considered in the differential diagnosis when increased Tl-201 activity is closely related to the joint. MRI aids in the differentiation by demonstrating features of hemosiderin degradation products. These findings are likely to be extremely helpful in the differential diagnosis of bone and soft tissue tumors.

## Introduction

Pigmented villonodular synovitis (PVNS) is a rare, idiopathic, proliferative disorder of the synovium (1) with two different forms: localized (LPVNS) and diffuse (DPVNS) (2). LPVNS occurs in small joints, whereas DPVNS typically affects the entire synovial lining of large joints. PVNS commonly occurs in patients aged 30–50 years and it has no gender bias (1). It is observed predominantly in the knee and hip and is less common in the ankle, shoulder and wrist (3,4). Elbow involvement is extremely rare; only 24 cases have been reported at this site (2–22). It is extremely difficult to differentiate PVNS from other soft tissue tumors on the basis of imaging alone. Therefore, biopsy is required for definitive diagnosis. We report a rare case of LPVNS arising from the elbow joint with contracture and discuss the findings of magnetic resonance imaging (MRI) and thallium (Tl)-201 scintigraphy, which were useful in making the differential diagnosis retrospectively.

## Case report

A 20-year-old, right-handed female reported a mass on the right elbow; this mass had caused progressive pain over the last 2 years. Although the patient had not suffered a previous trauma involving her right elbow joint, the patient recognized pain and a mass on the flexion side. Physical examination revealed an elastic and soft tumor measuring 3.0x3.0 cm. It demonstrated no adhesion to the skin and its margin was well-defined. The active range of motion of the affected elbow was −15° extension, 80° flexion, 90° supination and 90° pronation.

**Figure 1:**
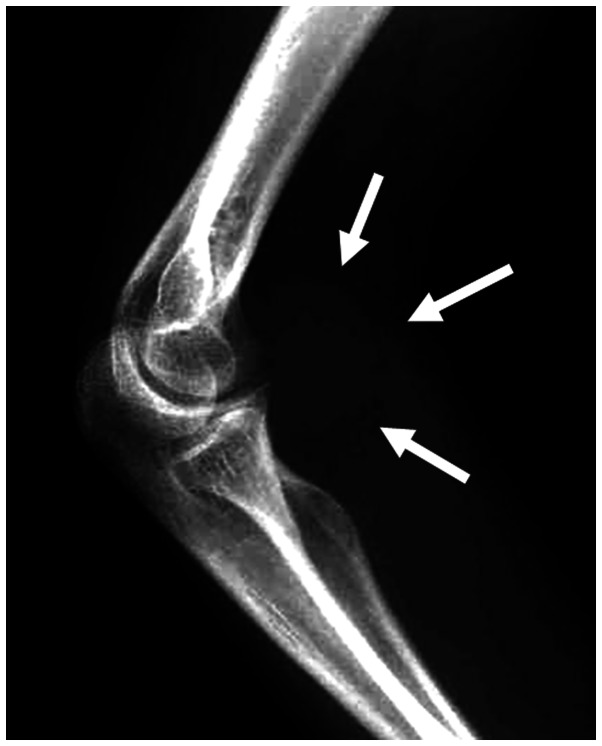
Radiograph showing a soft tissue mass (arrow) on the right elbow, with no apparent bone erosion.

**Figure 2:**
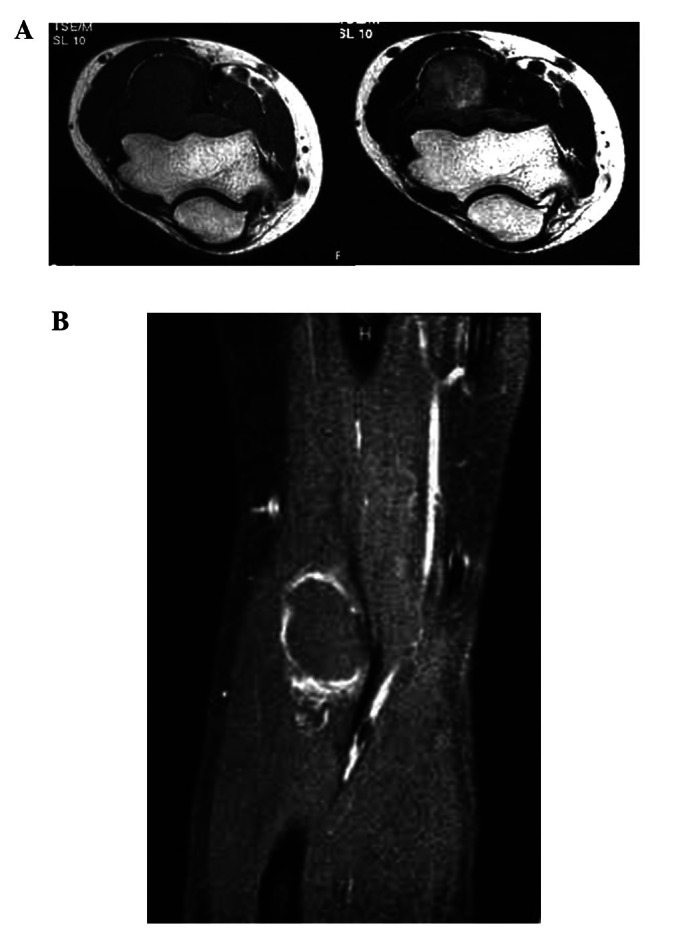
(A) Magnetic resonance image showing the tumor located between the brachioradialis and brachialis. The signal intensity of the tumor was isointense to muscle on T1-weighted images (left) and hyper- or isointense to muscle on T2-weighted images (right). (B) On gadolinium-enhanced magnetic resonance images, the margin of the tumor was well-contrasted.

**Figure 3:**
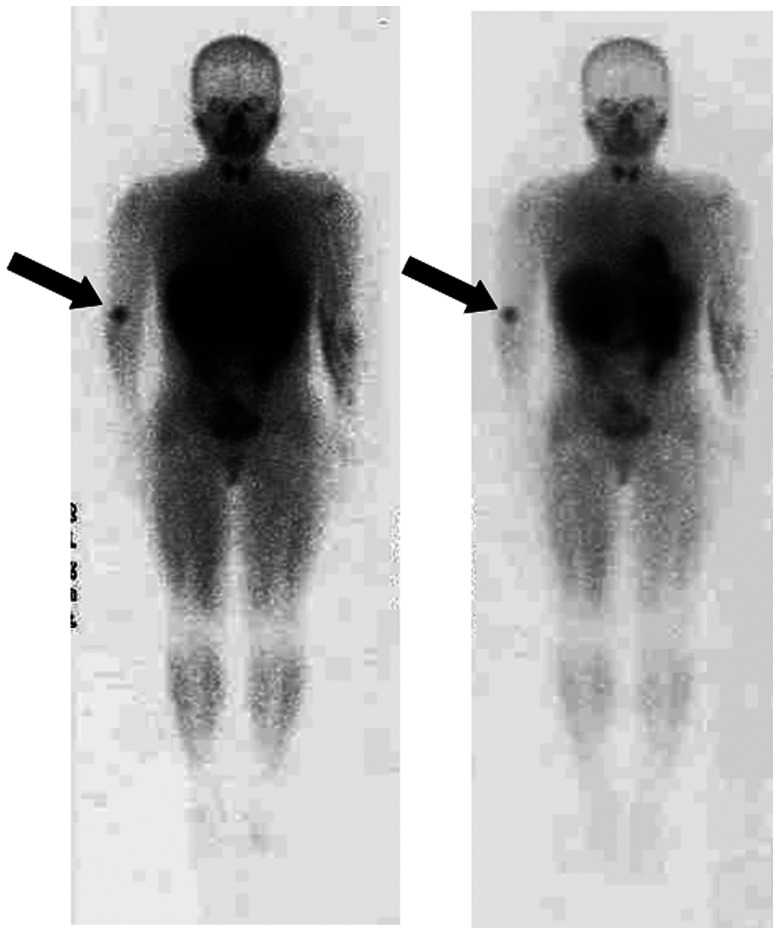
Thallium-201 scintigrams showing an abnormal accumulation (arrows) consistent with the tumor in the early (left) and delayed (right) phases. Such an abnormal accumulation was not observed at any other site.

A roentgenogram of the right elbow revealed a soft tissue mass around this joint, with no apparent bone erosion or cystic changes (Fig. 1). MRI revealed that the tumor was located between the brachioradialis and brachialis. The signal intensity of the tumor was isointense to muscle tissue on T1-weighted images; however, it was hyper- or isointense to muscle on T2-weighted images. In images obtained by gadolinium-enhanced MRI, the margin of the tumor was well-contrasted (Fig. 2A and B). Tl-201 scintigrams revealed an abnormal accumulation in the area of the mass in the early and delayed phases (Fig. 3). No other abnormal accumulation was detected.

**Figure 4:**
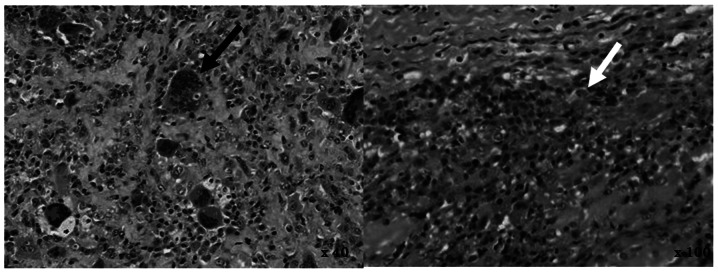
An open biopsy was performed. Microscopic examination revealed a nodular growth covered by synovial lining cells. Mitotic figures and a large number of multinucleated giant cells (black arrow, left) were observed in certain areas. Inflammatory cells were identified in various areas and the stroma demonstrated fibrosis. The white arrow indicates hemosiderin deposition (right). Histological findings were characteristic of pigmented villonodular synovitis.

On the basis of clinical findings and imaging characteristics, the tumor was diagnosed as a primary soft tissue tumor and an incisional biopsy was performed. Microscopic examination revealed a nodular growth covered by synovial lining cells. Mitotic figures were observed in parts, as were a large number of multinucleated giant and inflammatory cells. The stroma presented fibrosis and hemosiderin deposition was observed in areas of the surface (Fig. 4). Histological findings were characteristic of PVNS.

One month later, marginal excision with anterior capsulectomy was performed since the tumor demonstrated adhesion to the anterior lesion of the joint capsule. There was no apparent bone erosion and the capsule was brownish-yellow. The tumor was dumbbell shaped since a dull groove was created by the tendons of the brachioradialis and brachialis and its surface was smooth. Complete excision, including the anterior capsule was performed to reduce the risk of local recurrence. Following surgical treatment, the active range of motion of the operated joint was recovered fully to 0° extension and 140° flexion with physical training. The pain and mass were fully resolved following treatment and there was no recurrence 5 years after surgery.

## Discussion

The term PVNS was introduced by Jaffe *et al* in 1941, based on clinical and pathological experience (23). PVNS is a benign, locally invasive disease of the synovium and is currently classified as a giant cell tumor of the tendon sheath or diffuse-type giant cell tumor; the former description corresponds to LPVNS and the latter to DPVNS (24). LPVNS is a small, discrete lesion that usually occurs intra-articularly in the knee. The etiology of PVNS remains a matter of debate (6,7,11). Previous studies have not clarified whether the disease is a locally aggressive neoplasm or a reactive synovitis (4,5). It is clear, however, that the presence of blood in joints is essential for the occurrence of PVNS. Proliferation of villi occurs following hemorrhage in the joint space. A number of these villi are damaged and crushed by joint motion. Then, hemorrhage recurs and the synovium demonstrates continuous hyperplasia. The macroscopic appearance consists of a dark yellow-brown mass and villous thickening of the synovial membrane (7,19).

A literature review identified only 24 documented cases of PVNS involving the elbow joint; of these, 15 were case reports and nine were included in seven large retrospective PVNS series of all sites: one case from Scott (8), one from Granowitz *et al*(2), two from Docken (5), one from Pandey and Pandey (15), one from Miller (14), one from Ushijima *et al*(11) and two from Schwartz *et al*(4) (Table I). In the seven large retrospective PVNS series, LPVNS comprised 246 and DPVNS comprised 78 of the 421 cases. There were no details of the remaining 97 cases. In past reports of PVNS involving the elbow joint, five cases of LPVNS (18,19) and 17 cases of DPVNS were identified, in which this information was available. Gender was reported in 16 of 24 cases; there were four males and 12 females. Reported ages ranged from 6 to 61 years, with a mean age of 33.9 years in the 18 cases for which this information was available (2–22).

PVNS shows the same radiodensity as that of soft tissue. The disease causes contour erosion of the underlying bone or round lytic areas with well-demarcated borders, which are observed particularly at points of capsular insertion (17). MRI strongly supports the diagnosis of PVNS through the demonstration of areas of low- or iso-signal intensity on T1-and T2-weighted images, indicative of signal attenuation by the abnormal iron content in the hemosiderin within a thickened synovium (19,25). A number of studies have reported the MRI features of PVNS in the elbow joint (7,9,13,16,20). On T1-weighted images, the lesion demonstrates isointensity to muscle with small areas of low signal intensity, while T2-weighted images demonstrate mildly increased signal intensity in the lesion. Foci of low signal intensity are also demonstrated in similar regions on T2-weighted images. Furthermore, the lesion is enhanced heterogeneously by the administration of gadolinium-diethylene triamine pentaacetic acid (DTPA) contrast agent (9). These findings are different from those in malignant tumors, which show a mass of low- or iso-signal intensity on T1-weighted images and high signal intensity on T2-weighted images. Another classic feature observed within the synovium of other joints with PVNS is the presence of a fatty signal due to the accumulation of lipid-laden macrophages (7,17). However, this finding was not observed in our study.

It has been reported that PVNS demonstrates high isotope accumulation (26), although little has been reported about the scintigraphic findings of the disease (27). While a low retention rate of Tl-201 on delayed imaging is observed in benign tumors (28), Tl-201 uptake is observed on early and delayed images in almost all cases of PVNS (26,29); this simulates the findings of malignant disease (29). Given the above findings, the presence of activity on Tl-201 scintigraphy alone does not aid in the differentiation of PVNS from malignant diseases. However, this finding is useful in the differentiation of PVNS from other benign diseases. In addition, a diffuse nodular juxta-articular pattern of Tl-201 activity is strongly suggestive of PVNS.

In conclusion, we identified an extremely rare case of PVNS arising from the elbow joint. When interpreting Tl-201 images for the assessment of bone and soft tissue lesions, it is important to recognize PVNS as a condition that simulates malignant tumors. Furthermore, PVNS should be considered in the differential diagnosis when increased Tl-201 activity is closely related to the joint. MRI also aids in the differentiation by demonstrating features of hemosiderin degradation products. These findings are likely to be extremely helpful in the differential diagnosis of bone and soft tissue tumors.

## Figures and Tables

**Table I:** Literature review of pigmented villonodular synovitis (PVNS) arising from the elbow joint.

Sources	Knee	Hip	Ankle	Wrist	Elbow	Others	Total	Remarks
Scott (1968)	2	2			1		5	
Granowitz *et al*(1976)	20	2	4	6	1	62	95	
Docken (1979)					2		89	Incomplete data
Pandey and Pandey (1981)					1		47	Incomplete data
Miller (1982)	18	8	3	1	1	3	34	
Ushijima *et al*(1986)	25	5	13	2	1	6	52	
Schwartz *et al*(1989)	75	20			2	2	99	
Total	140	37	20	9	9	73	421	
